# Intranasal immunization with inactivated chlamydial elementary bodies formulated in VCG-chitosan nanoparticles induces robust immunity against intranasal *Chlamydia psittaci* challenge

**DOI:** 10.1038/s41598-021-89940-8

**Published:** 2021-05-17

**Authors:** Zonghui Zuo, Yongjuan Zou, Qiang Li, Yongxia Guo, Tianyuan Zhang, Jie Wu, Cheng He, Francis O. Eko

**Affiliations:** 1 Key Lab of Animal Epidemiology and Zoonosis, College of Veterinary Medicine, China Agricultural University, Beijing, 100193 People’s Republic of China; 2 Key Laboratory of Biopharmaceutical Production and Formulation Engineering, Chinese Academy of Sciences, Beijing, 100049 People’s Republic of China; 3 Department of Microbiology, Biochemistry and Immunology, Morehouse School of Medicine, Atlanta, GA 30310 USA

**Keywords:** Immunology, Microbiology

## Abstract

Vaccines based on live attenuated *Chlamydia* elementary bodies (EBs) can cause disease in vaccinated animals and the comparably safer inactivated whole EBs are only marginally protective. Recent studies show that a vaccine formulation comprising UV-inactivated EBs (EB) and appropriate mucosal delivery systems and/or adjuvants induced significant protective immunity. We tested the hypothesis that intranasal delivery of UV-inactivated *C. psittaci* EB formulated in *Vibrio cholerae* ghosts (VCG)-chitosan nanoparticles will induce protective immunity against intranasal challenge in SPF chickens. We first compared the impact of VCG and CpG adjuvants on protective immunity following IN mucosal and IM systemic delivery of EB formulated in chitosan hydrogel/microspheres. Immunologic analysis revealed that IN immunization in the presence of VCG induced higher levels of IFN-γ response than IM delivery or the CpG adjuvanted groups. Also, vaccine efficacy evaluation showed enhanced pharyngeal bacterial clearance and protection against lung lesions with the VCG adjuvanted vaccine formulation, thereby establishing the superior adjuvanticity of VCG over CpG. We next evaluated the impact of different concentrations of VCG on protective immunity following IN mucosal immunization. Interestingly, the adjuvanticity of VCG was concentration-dependent, since protective immunity induced following IN mucosal immunization showed dose-dependent immune responses and protection. These studies reveal that formulation of inactivated chlamydial antigens with adjuvants, such as VCG and chitosan increases their ability to induce protective immune responses against challenge.

## Introduction

Avian chlamydiosis or ornithosis is a widespread respiratory disease caused by the obligate intracellular zoonotic pathogen, *Chlamydia psittaci* and is a major cause of economic loss in the poultry industry^1–4^. Although infections are often systemic and can display severe, acute or chronic manifestations, most infections are asymptomatic^1,5^. Even when birds show no signs of infection, their productivity (meat and egg) can still be gravely affected^6,7^. In poultry flocks, transmission generally occurs through inhalation of aerosolized respiratory secretions or ingestion of contaminated dust and depending on the genotype and virulence of the C. *psittaci* strain involved (predominantly genotypes A and D), the infection can lead to pneumonia, air sacculitis, pericarditis, hepatitis and/or splenitis and death^1,8,9^. *C. psittaci* is also a permanent risk for zoonotic transmission to man (where it is referred to as psittacosis or parrot fever), leading to high morbidity and sometimes death^4,10^. Although effective antimicrobial therapies exist, the frequent recurrence of infections suggest that vaccination is likely the best approach to reduce the prevalence of avian chlamydial infections^11–13^.

Although differential susceptibility of different strains of *Chlamydia* to certain effectors have been reported^14^, similar immune effectors appear to control different species and serovars in animals and humans. It has been established that *Chlamydia* immunity is mediated primarily by a T helper type 1 (Th1) cell mediated immune (CMI) response requiring the induction and recruitment of IFN-γ -secreting CD4+ and CD8 + T cells^15^. Early studies revealed that unlike CD4 + T cells, elimination of CD8 + T cells does not compromise protection against a *C. trachomatis* genital infection^16^. On the hand, while in vivo depletion of CD8 + T cells in mice abrogated protection against *C. psittaci* infection*,* depletion of CD4 + T cells did not affect protection^17^ indicating that CD8 + T cells may play a more crucial role than CD4 + T cells in protection against *C. psittaci.* Although both T-cell-mediated and humoral immune responses are known to be elicited following chlamydial infection, a number of studies have demonstrated that the presence of antibodies during natural or experimental infection does not correlate with protection. Such studies include the findings that in vitro neutralizing antibodies do not transfer protective passive immunity to mice in vivo^18^ and the limited microbial clearance in experimental and clinical infections even in the presence of high levels of secretory and systemic antibodies^19–21^. These studies revealed that antibodies do not play a major role in protection during primary chlamydial infection. However, recent findings indicate that antibodies of the IgG2a and IgA isotypes enhance Th1 activation against chlamydiae^22–24^.

Initial efforts to develop *C. psittaci* vaccines were based on inactivated or attenuated chlamydial elementary bodies and were targeted at infections in companion cats and sheep^11,25–28^. However, inactivated vaccines although promising in principle, are only marginally protective^11^. Live attenuated vaccines utilizing live chlamydial yolk sac suspensions produced different levels of protection varying from near complete protection to none at all (cited in^25^). Also, vaccination with whole live organisms reduced the acute disease in experimentally infected cats but did not prevent shedding of the organism from the eye or on the transmission of infection to the gastrointestinal and genital tracts^28^, suggesting the need for alternative vaccine development strategies. A number of strategies have been employed in the effort to develop vaccines against *C. psittaci*. Most of these efforts were focused on vaccines based on the recombinant major outer membrane protein (MOMP), including MOMP-based DNA vaccines^29^, recombinant MOMP expressed by viral-vectors^30^ or a combination of DNA and MOMP^31^, and transgenic rice expressing MOMP^32^, and have been tested in avian or mouse models of *C. psittaci* infection. An analysis of the progress in *C. psittaci* vaccine development in poultry, including details of the type and efficacy of various experimental vaccines is the subject of a recent review^33^. Despite these concerted efforts however, there is still no effective licensed commercial vaccine against *C. psittaci* infection.

Immunization with live elementary bodies (EBs), the infectious form of *Chlamydia* has been shown to induce a significant degree of protective immunity marked by a lower intensity and shortened duration of infection but fails to protect against development of immunopathology^34,35^. UV-inactivated *Chlamydia* EBs (EB) on the other hand, do not provide significant protection against infection. The efficacy of such inactivated chlamydial vaccines can be improved by modifying the delivery vehicle and/or adjuvants, route of immunization or by using modified prime-boost vaccination protocols^36^. A recent study reported the induction of significant protective immunity similar to that induced by live chlamydial EBs, without the adverse pathologic consequences, following delivery of *C. trachomatis* EB with an appropriate adjuvant^37^. A number of adjuvants have been assessed for use in veterinary vaccines, including aluminum, Montanide, biodegradable polymeric microparticles, and nanoparticles^38^.

Nanoparticles (NPs) have in recent times been of increasing interest in various fields, including their utility as adjuvants and delivery systems in vaccine development. Previous studies have shown that chitosan NPs are promising delivery systems for enhancing vaccine efficacy^39,40^. Chitosan possesses well-defined properties that include biocompatibility, low cost^41^, high safety profile, and the capacity to stimulate both antibody and robust T helper type-1 (Th1) responses^42,43^. Chitosan has been demonstrated to have distinct mucoadhesive properties, which prolongs antigen retention at mucosal sites^44^. Moreover, the versatility of chitosan enables a diversity of formulations for chitosan-based delivery systems, such as solutions, powders, micro/nanoparticles, hydrogels, and microneedle patches, to meet the various needs of novel vaccination methods^45,46^. Besides, the *Vibrio cholerae* ghost (VCG) platform has been shown to be an effective delivery/adjuvant system for cloned antigens inducing immune responses and protection even in the absence of external adjuvants^47–49^. VCG are empty bacterial cell envelopes devoid of cytoplasmic contents and cholera toxin and are produced by genetic inactivation of *V. cholerae* cells, involving the controlled expression of cloned bacteriophage PhiX174 lysis gene *E*. The resulting cell envelopes (VCG) share the functional and antigenic determinants of the envelope with their living counterparts^47^. Unmethylated CpG motifs, the agonists of Toll-like receptor (TLR) 9 or TLR 21 in chickens, are pathogen-associated molecular patterns and well-known stimulators of Th1 immune responses. We previously showed that CpG ODN enhanced the cellular immune responses to an OmpA-based *C. psittaci* DNA vaccine following intramuscular immunization of SPF chicken^50^. In other studies, CpG ODN was shown to enhance the efficacy of *C. trachomatis* and *C. muridarum* subunit antigens in mice^51,52^. In the current study, we tested the ability of *C. psittaci* EB formulated in VCG-chitosan nanoparticles to induce protective immunity against intranasal challenge in SPF chickens. We demonstrated the superiority of mucosal over systemic route for delivery of *C. psittaci* EB. The study also revealed that formulation of inactivated chlamydial antigens with VCG enhanced their immunogenicity and protective efficacy in a dose dependent manner.

## Methods

### Ethics statement

This study was carried out in strict accordance with the recommendations in the Regulations for the Administration of Affairs Concerning Experimental Animals of the State Council of the People’s Republic of China. The study protocol was approved by the Committee on Experimental. Animal Management of China Agricultural University.

### *Chlamydia psittaci* strains and antigens

*C. psittaci* strain 6BC (ATCC VR-125, genotype A) and *C. psittaci* strain HJ (genotype A, identified by Alere DNA Microarray^53^, originally isolated from the lungs of a meat pigeon with respiratory distress^54^. Stock preparations were generated by propagating live EBs in Buffalo Green Monkey Kidney (BGMK) cells^55^. EBs were purified by density gradient centrifugation with gastrografin (meglumine diatrizoate)^56^ and titers (number of inclusion forming units (IFUs)/ml were determined by an immunofluorescence assay described previously^55^. *C. psittaci* antigen was prepared by UV-inactivation of 1 × 10^6^ IFU of strain 6BC EBs. Aliquots of purified EB suspensions in Eppendorf tubes were exposed to a 15 W UV lamp positioned at a distance of 20 cm from the tube surface^37^ for 1 h. To confirm complete inactivation, fresh Buffalo green monkey kidney (BGMK) cells were infected with the UV-inactivated EBs for 48 h, washed three times with PBS and fixed with ice-cold methanol. The cells were then stained with fluorescence-labeled anti-MOMP monoclonal-antibody (previously prepared in our lab) and examined under the microscope to confirm the presence or absence of chlamydial inclusions. *C. psittaci* strain 6BC was used for immunization while strain HJ was used in challenge studies.

### Preparation of vaccine formulations

*Vibrio cholerae* ghosts (VCG) were produced by genetic inactivation (protein *E*-mediated lysis) of *Vibrio cholerae* cells essentially as described previously^57^. This essentially involves introduction by protein E of a lysis tunnel structure through the cell envelope complex, which leads to expulsion of the entire cytoplasmic material from the cells, resulting in empty cell envelopes. The cell envelopes (VCG) were then harvested by centrifugation, washed several times with a low ionic buffer, freeze-dried and stored refrigerated until used. CpG oligonucleotide DNA (5’-TCGTCGAAGTCGTTTTGGGGGG-3’) was synthesized by Sangon Biotech (Shanghai, China)^58^.

The novel thermosensitive hydrogel composed of the cationically modified chitosan, N-(2-hydroxypropyl)-3-trimethylammonium chitosan chloride (HTCC) and α-β-glycerophosphate (α-β-GP) constituted the vaccine delivery system. A 10 ml chitosan hydrogel solution was formulated by mixing HTCC (0.26 g), sodium glycerophosphate (1.8 g), 5 ml lactic acid (0.15 M) and 5 ml deionized water at room temperature as previously described^46^. The vaccines were formulated by adding *C. psittaci* 6BC EB (10^6^ IFU) in the presence or absence of varying concentrations of VCG (25 μg, 35 μg, 50 μg or 100 μg) or 35 μg CpG adjuvant into the chitosan hydrogel solution at a ratio of 1:1 and stirred gently to blend completely. This resulted in a clear homogeneous HTCC hydrogel solution at room temperature. The hydrogel was then processed into positively charged microspheres by membrane emulsification at 37 °C as previously described^59^. Each microsphere, with an average particle diameter of 1979 nm determined as previously reported^60^, has a pore diameter of 1000 nm enabling the encapsulation of negatively charged EB (diameter, 300–800 nm) and adjuvants. The vaccine formulations were transferred into sterile tubes and stored at 4 °C until used. The liquid formulation at room temperature was used for intranasal (IN) administration while the hydrogel microspheres formulation was employed for intramuscular (IM) delivery.

### Chickens

A total of 233 7-day-old SPF chickens (Weitong Merial Laboratory Animal Co., Beijing, China) used in this study were maintained in Horsfall-type isolators in biosafety level 2 facilities in the Animal Experimental Center of China Agricultural University. The study was carried out in strict accordance with the recommendations in the Regulations for the Administration of Affairs Concerning Experimental Animals of the State Council of the People’s Republic of China. The study protocol was approved by the Committee on Experimental Animal Management of China Agricultural University. All immunization and challenge procedures were performed without anesthesia but under humane conditions and efforts were made to minimize suffering. All animals were euthanized in a CO_2_ chamber using 100% CO_2_ at a flow rate of 10–30% of the Chamber Volume per Minute (CV/min) followed by cervical dislocation. We also confirm that the study was carried out in compliance with the ARRIVE guidelines.

### Experimental design and immunization protocol

In a preliminary study, we compared the ability of chitosan Gel, VCG and CpG to enhance the immune responses and protection induced following IN delivery of EB. Thus, three groups of 7-day-old SPF chickens were immunized IN (10/group) and boosted 2 weeks apart with 10^6^ IFU of EB in combination with 50 μg chitosan gel (Gel + EB) or 35 μg of VCG (VCG + EB) or 35 μg of CpG (CpG + EB). MOMP-specific antibodies in serum samples obtained on days 7, 14 and 21 post primary immunization (when chickens were 14, 21 and 28 days old, respectively) were measured by antibody ELISA described below. Levels of cytokines in lung lavage fluid obtained 2 weeks post booster immunization were evaluated by cytokine ELISA. Also, immunized chickens were challenged intranasally 2 weeks post booster immunization (when chickens were 36 days old) with 1 × 10^8^ IFU of live *C. psittaci* HJ EBs and the lungs and air sacs were examined for pathological lesions and scored 12 days post challenge as described below.

**Figure 1:**
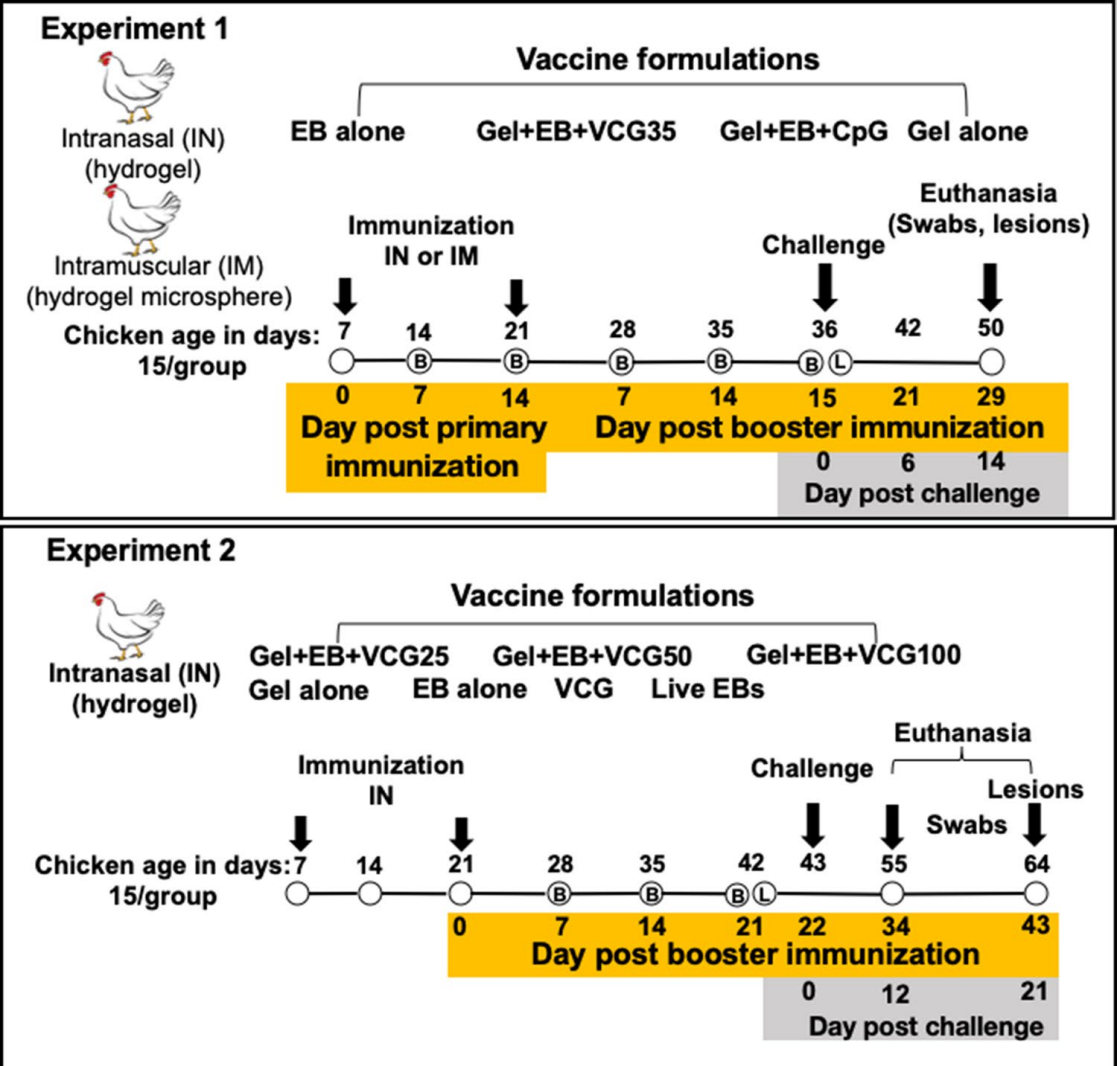
Schematic diagram of the experimental design outlining the immunization, challenge, and sample collection schedules. B, Blood samples; L, Lung lavage samples.

The main study was performed in two parts according to the experimental design shown in Fig. 1. First, we compared the adjuvant capacity of VCG with CpG in enhancing the immune responses and protection induced by mucosal and systemic delivery of EB using chitosan gel as a carrier/delivery system. Next, we evaluated the protective immune responses generated by immunization with EB co-delivered with different concentrations of VCG. In Experiment 1, 7-day-old SPF chickens were immunized IN (15/group) or IM (15/group) twice, 2 weeks apart with 10^6^ IFU of EB alone or the appropriate chitosan hydrogel formulation incorporating EB (10^6^ IFU) and either 35 μg of VCG (Gel-EB-VCG35) or 35 μg of CpG (Gel-EB-CpG). A sham immunized group received 50 μg of the chitosan Gel alone. In Experiment 2, 7-day-old SPF chickens were immunized IN (15/group) twice, 2 weeks apart with 10^6^ IFU of EB alone or with the chitosan hydrogel formulation incorporating EB (10^6^ IFU) together with either 25 μg or 50 μg or 100 μg of VCG. A group of chickens (15/group) that received a single IN dose of live *C. psittaci* 6BC EBs (1 × 10^5^) served as positive control while groups that received 50 μg of VCG or 50 μg chitosan hydrogel alone served as negative controls.

### Collection of lung lavage fluid

Immunized and control chickens (6/group) were euthanized by CO_2_ inhalation 2 or 3 weeks post booster immunization (Fig. 1) and lung lavage fluids were harvested using a lung lavage procedure, which permits simple, rapid collection of lung samples with minimal labor as described previously^61^. Briefly, a lung lavage device consisting of two TygonJ tubing with unequal diameters (Fisher Scientific, Norcross, GA) was attached to a syringe containing 5 ml of lavage solution (1 M Tris/glycine buffer with 0.25% Tween 20, pH 7–8). Collection of lavage fluid involved inserting the narrow diameter tubing down the trachea, slowly administering 5 ml of PBS into the lungs and withdrawing about 3 ml of lavage fluid. The samples were centrifuged at 1000 g for 10 min at 4 °C to remove debris and the supernatants were collected and stored at −80 °C until analyzed.

### Cytokine ELISA assays

The magnitude of cytokines in lung lavage fluids was assessed using the IFN-γ (PB0442C-100, IL-2 (PB0387C-100), IL-12 (PB0435C-100), IL-10 (KP1116C-100)] ELISA kits from Kingfisher (Kingfisher Biotech Inc, Saint Paul, MN)^62^. For each cytokine, the recombinant protein, capture antibody, and detection antibody were obtained as a complete kit and the protocol was optimized for each ELISA (Supp Table 1). Plates were coated overnight at room temperature with Capture Antibody in Dulbecco’s Phosphate Buffered Saline (DPBS) containing 4% BSA. Nonspecific binding sites were blocked for 3 h at room temperature with 100 μl of 1% non-fat dry milk (ThermoFisher Life Sciences, Shanghai, China). For all ELISA assays, 100 μl of lung lavage fluid or recombinant IFN-γ, IL-2, IL-12, and IL-10 protein standards were added to appropriate wells and incubated at room temperature for 1 h. The limit of detection for the cytokine ELISAs was determined to be 31.25 pg/ml. Plates were incubated with 100 μl of Chicken Biotinylated Polyclonal Antibody for 1 h and 100 μl of Streptavidin-HRP solution (AR0068-001) for 30 min. The plates were developed by incubation with 100 μl of TMB substrate solution (AR0133-002) in the dark at room temperature for 30 min and 100 μl of TMB Stop Solution was added to stop the reaction. The absorbance was read at 450 nm on a universal Microplate Reader (ThermoFisher Life Sciences, Shanghai, China). Dilutions for protein standards, detection antibody, and streptavidin-HRP were in DPBS containing 4% BSA. All washes were done using PBS containing 0.05% Tween-20. Levels of the Th1 (IL-18, #CSB-E10070Ch) and Th2 (IL-4, # CSB-E06756Ch) cytokines were assessed using commercial ELISA kits from Cusabio (Cusabio, Wuhan, China) according to the manufacturer’s instructions; the detection ranges were 7.8–500 pg/ml and 12.5–800 pg/ml, respectively. Briefly, 100 μl of lung lavage fluid or recombinant IL-18 or IL-4 protein standards was added to appropriate wells of microplates pre-coated with antibody specific for IL-18 or IL-4 and incubated at 37 °C for 2 h. After removing the liquid from each well (without washing), 100 μl of biotin-conjugated antibody specific for IL-18 or IL-4 were added to each well and incubated for 1 h at 37 °C. After washing, 100 μl of avidin-HRP was added to the wells and incubated for 1 h at 37 °C. Following another wash to remove any unbound avidin-enzyme reagent, 90 μl of TMB substrate solution was added to each well and incubated for 30 min at 37 °C protected from light. Color development was stopped by addition of 50 μl of Stop Solution and the absorbance was read at 450 nm as indicated above. All assays were performed in triplicates and were repeated for validation. Data were calculated as mean cytokine concentrations (± S.D.) in samples from 6 chickens in each group and is representative of one of two independent assays with similar results.

### Measurement of lymphocyte proliferation

We used the 5-Bromo-2’-deoxy-uridine (BrdU) cell proliferation assay (ab126556; Abcam, Beijing, China) to evaluate the ability of the various vaccine formulations to elicit T cell proliferation essentially according to the manufacturer’s protocol with slight modifications as described previously^48^. Peripheral blood mononuclear cells (PBMCs) were isolated from heparinized blood samples collected from immunized and control chickens (6/group) by Lymphoprep density gradient centrifugation (Solarbio Science & Technology, Beijing, China) 2 weeks (Experiment 1) or 3 weeks (Experiment 2) post booster immunization. Samples were incubated in 96-well flat bottom plates (1 × 10^6^ cells/well in RAPI medium containing 2% chicken serum) for 24 h. The cells were restimulated with 10^6^ IFU of UV-inactivated *C. psittaci* EBs (*C. psittaci* antigen) for 24 h at 37℃ in 5% CO_2_. Control cultures containing PBMCs and medium without antigen (unstimulated) served as internal background control. After stimulation, cells were incubated with BrdU for 12 h and fixing/denaturing solution was added for 30 min at room temperature. After removal of the solution, anti-BrdU monoclonal Detector antibody was added to the cells for 1 h at room temperature, washed and incubated with HRP-conjugated goat anti-mouse IgG antibody for 30 min at room temperature. TMB substrate was then added for 30 min at room temperature in the dark. Stop Solution was added to stop the reaction and the optical density of the colored reaction product (bright yellow) was read at 450 nm on a universal Microplate Reader (ThermoFisher Life Sciences, Shanghai, China). Data were calculated as the mean values (± S.D.) for triplicate cultures for each experiment.

### Determination of MOMP-specific serum antibody levels by ELISA

Blood samples were collected via the ulnar vein on days 7 and 14 after the primary immunization, and again on days 7 and 14 after the booster dose (Experiment 1). In Experiment 2, blood samples were obtained on days 7, 14 and 21 after the booster immunization. Sera were obtained by centrifugation at 1500 g for 8 min and stored at − 20 ℃ until used. Negative control sera were obtained from naive 7-day-old SPF chickens. Positive control sera were obtained from previously experimentally infected chickens. Serum antibody titers were analyzed by a recombinant MOMP-based ELISA assay described previously^63^ using a twofold dilution series starting at a dilution of 1 in 40. Recombinant MOMP was produced in pcDNA1/MOMP-transfected COS7 cells as described previously^64^. Briefly, ELISA plates were coated with *C. psittaci* rMOMP (2 μg/ml; 100 μl/well of a 96-well plate) and incubated with the serum dilutions. After washing, biotin labeled goat anti-chicken/turkey IgG (H + L) antibody conjugate (Nordic, Tilburg, The Netherlands) diluted 1:2000 was added. The plate was washed after 1 h and then incubated for another hour with peroxidase-conjugated streptavidin diluted 1:4000. After washing, ABTS substrate (2,2' azinodi-3-ethylbenzothiazoline sulphonate, Bommeli AG) was added to the wells for 30 min and the absorbances were read at 450 nm on a universal Microplate Reader (ThermoFisher Life Sciences, Shanghai, China). Anti-MOMP antibody titers were determined as the highest serum dilution that gave an optical density (OD_450_) above the cut-off value, which was set as the mean of the OD values of negative chicken sera plus two times the standard deviation.

### Intranasal challenge and determination of pharyngeal *C. psittaci* burden

Immunized chickens were challenged intranasally 2 weeks (Experiment 1) or 3 weeks (Experiment 2) after the booster immunization with 1 × 10^8^ IFU of live *C. psittaci* strain HJ EBs in 20 μl of SPG (sucrose-phosphate-glutamic acid (75 g/L sucrose, 0.52 g/L KH_2_PO_4_, 1.22 g/L Na_2_HPO_4_, 0.72 g/L L-Glutamic acid, pH 7.4) buffer. Chickens were observed daily for clinical signs, such as runny nose, breathing difficulty and lethargy to establish their health status. Chickens from each group were euthanized 2 weeks (Experiments 1 and 2) and at 3 weeks (Experiment 2) post challenge and pharyngeal swabs were obtained from individual chickens in each group and stored in SPG buffer and subsequently inoculated onto BGMK cell monolayers. Intracellular *C. psittaci* inclusions in pharyngeal swabs were detected by fluorescence microscopy using the Imagen immunofluorescence kit (Oxoid, UK) as described previously^10,31^.

### Evaluation of lung/air sac pathological lesions

Lungs and air sacs were harvested from each group of euthanized chickens (above), examined for pathological lesions and scored. Lesions were recorded as either absent or present. If absent (i.e. normal), a score of 0 was assigned. If present, the severity of the lesions was recorded as mild, moderate, or severe, with pathology lesion severity scores of 1 through 3 for lungs, thoracic air sacs and abdominal air sacs. Lesion severity scores were defined as follows. For air sacs; thoracic air sacs and abdominal air sacs respectively: 0, no significant tissue alterations (thin and transparent air sacs); 1, slightly thickened and opaque air sacs; 2, obviously thickened air sacs with exudations; 3, presence of yellowish cellulose discharge. For lungs: 0, 14, lines normal; 1, 30% of lung surface area showed hemorrhage, degeneration or necrosis; 2, 60% of lung surface area showed hemorrhage, degeneration or necrosis; 3, entire lung surface showed hemorrhage, degeneration or necrosis. Based on these criteria, the calculated total lesion severity score, which is the sum of the scores for lungs, thoracic air sacs and abdominal air sacs were recorded for each chicken.

### Statistical analysis

The GraphPad Prism 8.0 software (GraphPad Software, Inc., La Jolla, CA) and IBM SPSS 26.0 software were used to perform statistical analysis on a Mac computer. Data distribution was calculated with Kolmogorov–Smirnov test and Q-Q plot. Data fitted to normal distribution (stimulation index, cytokine levels, chlamydial shedding, antibody titers and gross lesion scores) were analyzed with one-way analysis of variance (ANOVA) with Tukey’s post multiple comparison test. Statistical significance was determined at probability (p) values ≤ 0.05, 0.01 or 0.001.

## Results

### VCG was most effective compared to chitosan Gel or CpG in protecting against lung lesions following IN mucosal immunization with EB

In the preliminary study, the cytokine levels induced by Gel + EB, VCG + EB and CpG + EB are shown in Supp Fig. 1. IFN-γ levels (A) induced by the VCG + EB formulation was significantly higher (*p* < 0.05) compared to Gel + EB and CpG + EB groups, which were comparable. Also, levels of IL-12 (B) induced by the VCG + EB were significantly higher (*p* < 0.001) compared to Gel + EB and CpG + EB. However, while VCG + EB-induced IL-2 levels (C) were significantly higher (*p* < 0.01) compared to Gel + EB, they were comparable to those of CpG + EB. On the other hand, levels of the anti-inflammatory cytokine IL-10 (D) were lowest in the Gel + EB and highest in the CpG + EB group. The antibody ELISA results showed Gel + EB elicited significantly higher (*p* < 0.05) absorbance values (OD_450_) compared to CpG + EB on day 7 post primary immunization. On day 14, while absorbance values of Gel + EB and VCG + EB were comparable, they were significantly higher (*p* < 0.05) compared to CpG + EB. By day 21, however absorbance values in all groups were comparable (Supp Fig. 2). The combined pathological lesion scores of lungs and air sacs of chickens evaluated 2 weeks after challenge showed VCG + EB-immunized chickens had significantly reduced lesions compared to those in the Gel + EB (*p* < 0.05) and CpG + EB (*p* < 0.01) immunized groups, which showed more severe pathological lesions (Supp Fig. 3).

**Figure 2:**
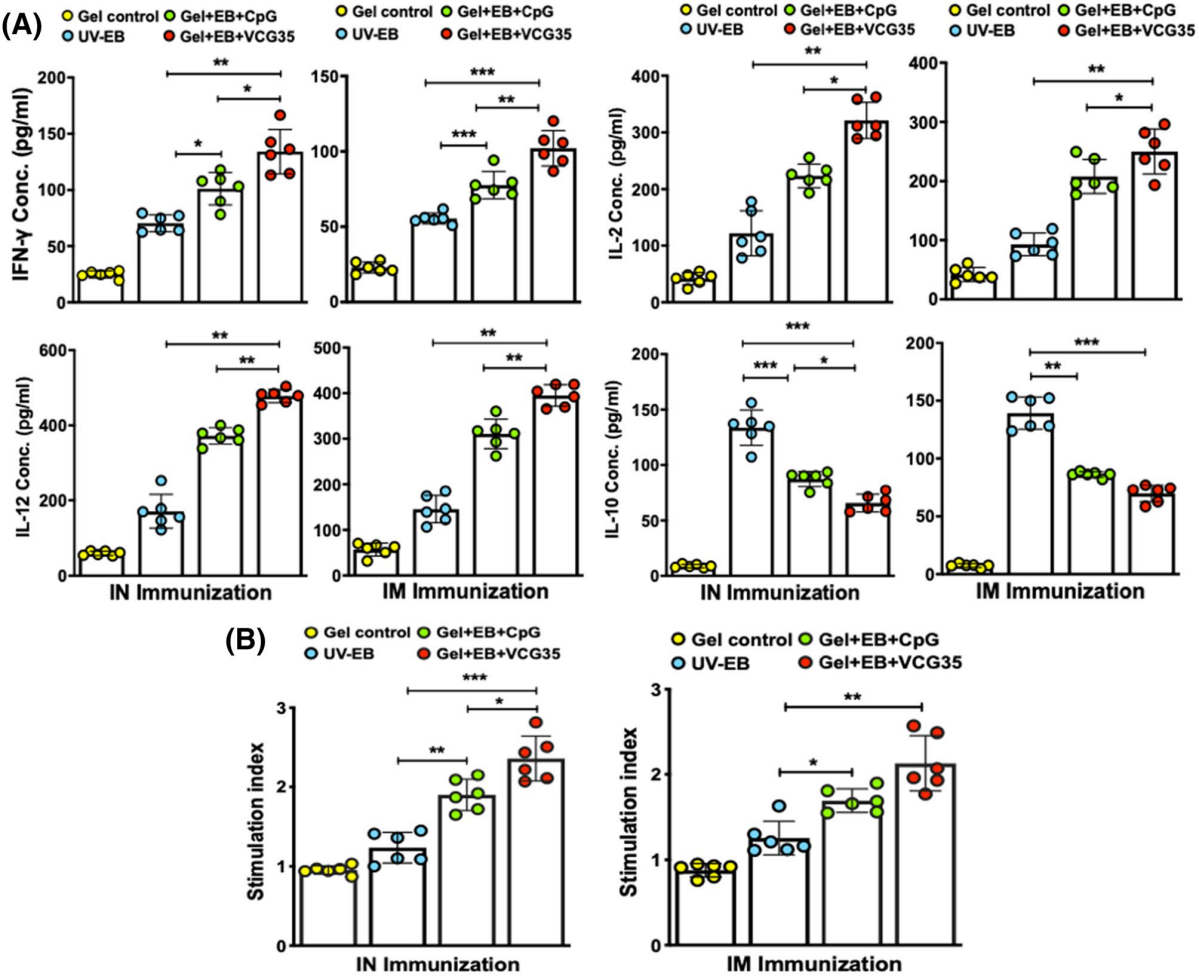
Cytokine levels in lung lavage fluid and *Chlamydia psittaci*-specific proliferative responses of peripheral blood mononuclear cells (PBMCs). Groups of chickens were immunized and boosted IN or IM as described in the materials and methods section. (**A**) Two weeks after the booster immunization, the amount of IFN-γ, IL-2, IL-12 and IL-10 cytokines contained in lung lavage fluids from chickens immunized IN or IM (6/group) was measured using single cytokine assay kits. The concentration of the cytokines in each sample was obtained by extrapolation from a standard calibration curve generated simultaneously. Each cytokine ELISA assay was performed twice with similar results. The data shown is one of those two assays and the bars represent the mean cytokine concentrations (± S.D.) in samples from six chickens in each group. (**B**) PBMCs obtained from six chickens/group by Lymphoprep density gradient centrifugation 2 weeks after the booster immunization were restimulated in vitro with 10^6^ IFU of UV-irradiated *C. psittaci* EBs (*C. psittaci* antigen) for 24 h at 37℃ in 5% CO_2_. Antigen-specific proliferative responses were determined using the 5-Bromo-2’-deoxy-uridine (BrdU) cell proliferation assay kit (Abcam, Beijing, China) as described in the Materials and Methods section. BrdU incorporation was detected by addition of anti-BrdU antibody and the absorbance was read at 450 nm. Results are expressed as the stimulation index (SI), the ratio between absorbance values of stimulated and non-stimulated cells. The bars represent the mean SI values (± S.D.) of samples from six chickens in each group and the data shown is one of two independent lymphoproliferative assays with similar results. Significant differences between groups were evaluated by One-way ANOVA with Tukey’s post multiple comparison test at p* < 0.05, p* < 0.01 and p*** < 0.001).

**Figure 3:**
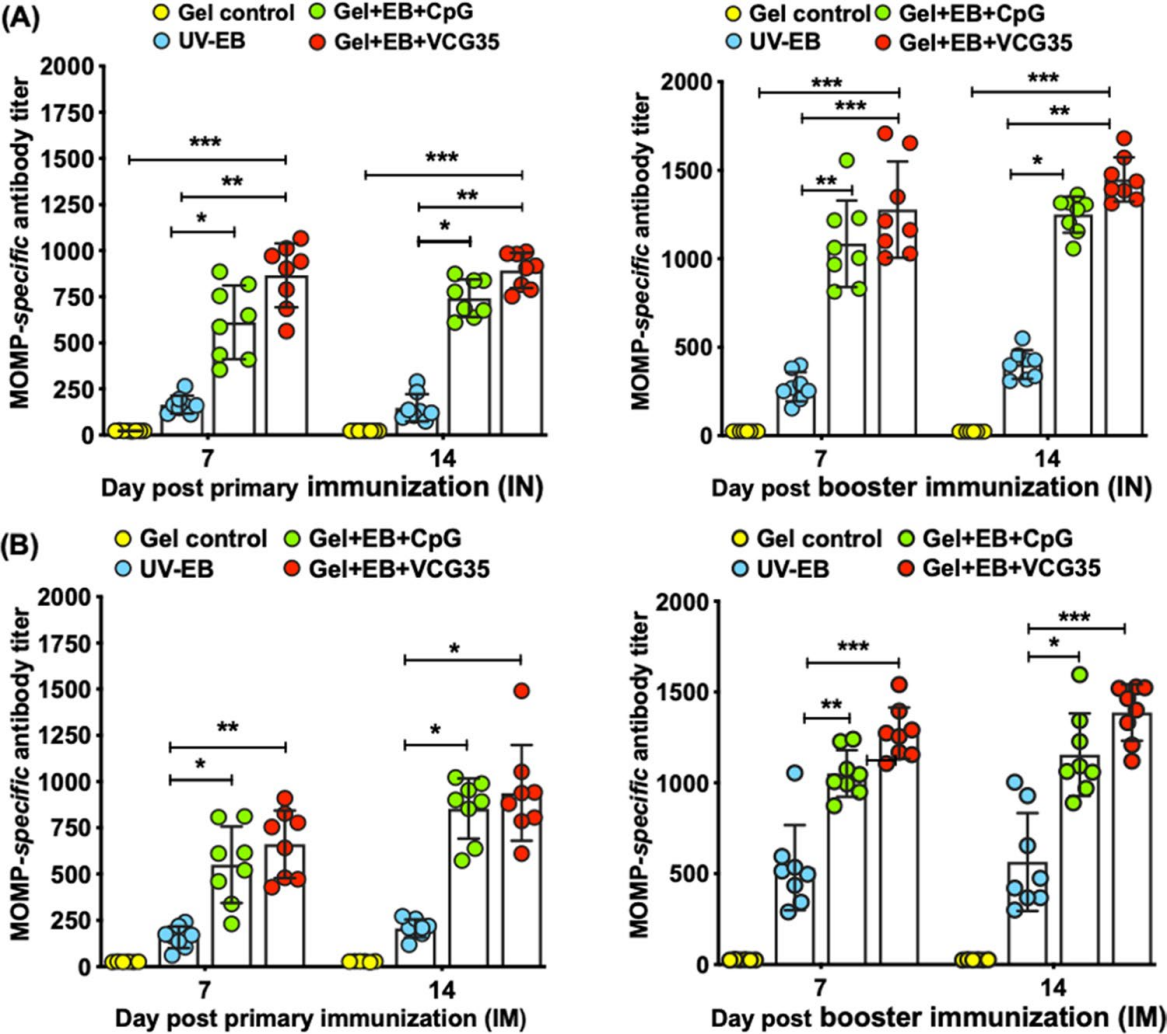
MOMP-specific antibody titers elicited after immunization. Groups of chickens were immunized IN or IM twice, 2 weeks apart as described above. Serum samples were obtained from 8 chickens/group on days 7 and 14 after the primary immunization, and again on days 7 and 14 after the booster dose. A MOMP-based antibody ELISA with twofold serum dilutions were used to assess the titer of antibodies elicited in serum after IN (**A**) and IM (**B**) immunization. Antibody titers were determined as the highest serum dilution that gave an optical density (OD_450_) above the cut-off value (mean negative OD values plus 2-SD). Results (one of two independent experiments with similar results) are presented as the reciprocal of the highest serum dilution with an absorbance above the cut-off value. Significant differences between experimental groups were evaluated by One-way ANOVA with Tukey’s post multiple comparison test at (p* < 0.01).

### Induction of antigen-specific cytokine responses

Two weeks after the booster immunization, both IN and IM routes of immunization stimulated the production in chicken lung lavage fluid of significantly higher levels of Th1-type cytokines IFN-γ, IL-2 and IL-12 compared to EB alone or Gel alone (control), with the VCG-adjuvanted group showing an immunogenic advantage (*p* < 0.01) (Fig. 2A). Also, the Gel + EB + VCG35 vaccine induced significantly higher levels of Th1-type cytokines with varying levels of significance compared to the Gel + EB + CpG vaccine, irrespective of route of immunization. Furthermore, the amounts of Th1 cytokines induced by the Gel + EB + VCG35 vaccine was significantly higher (p < 0.05) in IN immunized chickens compared to chickens immunized by the IM route. On the other hand, the amount of IL-10 elicited in lung lavage fluid from EB-immunized chickens was significantly higher (*p* < 0.01) compared to EB combined with either VCG or CpG, irrespective of route of immunization. In general, these levels were comparable following IN mucosal and IM systemic immunization.

### Lymphocyte proliferative responses of chicken after IN and IM immunization

The *C. psittaci*-specific proliferative responses elicited 2 weeks after the booster immunization were compared by analyzing the amount of BrdU incorporation following stimulation of PBMCs with or without *C. psittaci* antigen. The magnitude of T cell proliferation was expressed as stimulation index (SI), defined as the ratio of the absorbance values of stimulated and non-stimulated cells. Regardless of route of vaccine delivery, PBMCs from chickens immunized with EB together with CpG or VCG elicited significantly higher (*p* < 0.01) proliferative responses to antigenic restimulation compared to EB or Gel alone (control) (Fig. 2B). SI values obtained following both delivery routes were comparable. Moreover, co-delivery of EB with VCG generated significantly higher (*p* < 0.05) proliferative responses compared to CpG following IN but not IM delivery.

### Induction of MOMP-specific antibodies in serum following IN and IM immunization

Following the primary immunization dose, both IN and IM immunization with EB in combination with either CpG or VCG elicited significantly higher (*p* < 0.01) MOMP-specific serum antibodies compared to EB or Gel alone (control) (Fig. 3). Administration of the booster dose further enhanced antibody titers 7- and 14-days post boost. Additionally, the impact of co-delivery of EB with VCG was not significantly different from that of CpG, irrespective of delivery method.

### Reduction in pharyngeal chlamydial shedding in mucosally-immunized chickens infected with *C. psittaci*

**Figure 4:**
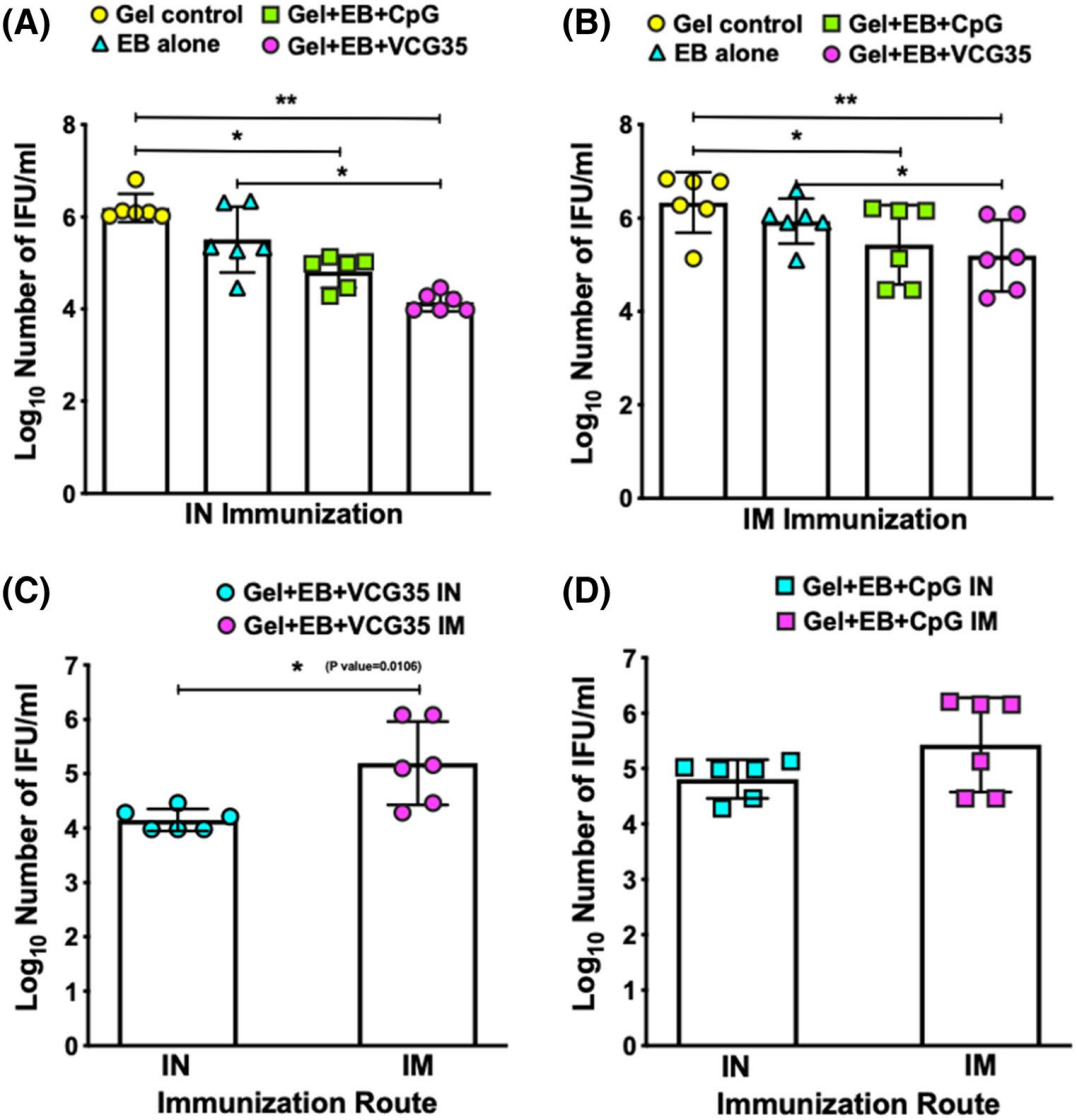
Pharyngeal chlamydial burden in immunized chickens challenged with *C. psittaci*. Groups of chickens were immunized IN (**A**) or IM (**B**) as described above and challenged intranasally with 1 × 10^8^ IFU of live *C. psittaci* 2 weeks after the booster immunization. To monitor bacterial burden, pharyngeal swabs were obtained from individual chickens (6/group) 2 weeks post challenge and *C. psittaci* was isolated from swabs in tissue culture. Chlamydial inclusions were detected by fluorescence microscopy using the Imagen immunofluorescence kit (Oxoid, UK) and enumerated. The data show the number of recoverable IFUs expressed as log_10_ IFU/ml. Comparison of the number of recoverable IFUs shed by Gel + EB + VCG35 (**C**) and Gel + EB + CpG (D) following IN and IM administration is also shown. Differences between experimental groups within each administration route were compared by One-way ANOVA with Tukey’s post multiple comparison test while differences in number of recoverable IFUs between IN and IM administration were compared by 2-tailed t-test at p* < 0.05 and p** < 0.01.

Two weeks post challenge, the pharyngeal *C. psittaci* load in chickens immunized IN with the VCG-adjuvanted vaccine formulation (Gel + EB + VCG35) was significantly lower compared to the Gel control (*p* < 0.01) and EB (*p* < 0.05) groups (Fig. 4A). These chickens shed about 1.4-log and approximately 1-log higher IFUs, respectively compared to the Gel + EB + VCG35 vaccine group. The difference between the *C. psittaci* load in Gel + EB + CpG immunized chickens was significant (*p* < 0.05) when compared to the Gel control group. Although chickens immunized IN with the Gel + EB + VCG35 vaccine shed fewer *C. psittaci* IFUs compared to the Gel + EB + CpG vaccine group, the difference was not statistically significant (*p* > 0.05). Among the IM immunized chickens, the Gel + EB + VCG35 and Gel + EB + CpG vaccine groups shed significantly fewer (*p* < 0.05) *C. psittaci* IFUs than chickens in the Gel control group (Fig. 4B). Furthermore, chickens immunized IN with the Gel + EB + VCG35 vaccine shed significantly fewer (*p* < 0.05) *C. psittaci* IFUs (Log_10_ 4.15) compared to those immunized IM (Log_10_ 5.19) (Fig. 4C) while *C. psittaci* shedding in chicken immunized with Gel + EB + CpG was comparable between IN and IM (Fig. 4D). Focusing on the Gel + EB + VCG35 vaccine, the results indicate the IN mucosal route was superior to the IM route in conferring protection against pharyngeal chlamydial shedding.

### Comparison of lung and air sac lesions in immunized chickens infected with *C. psittaci*

**Figure 5:**
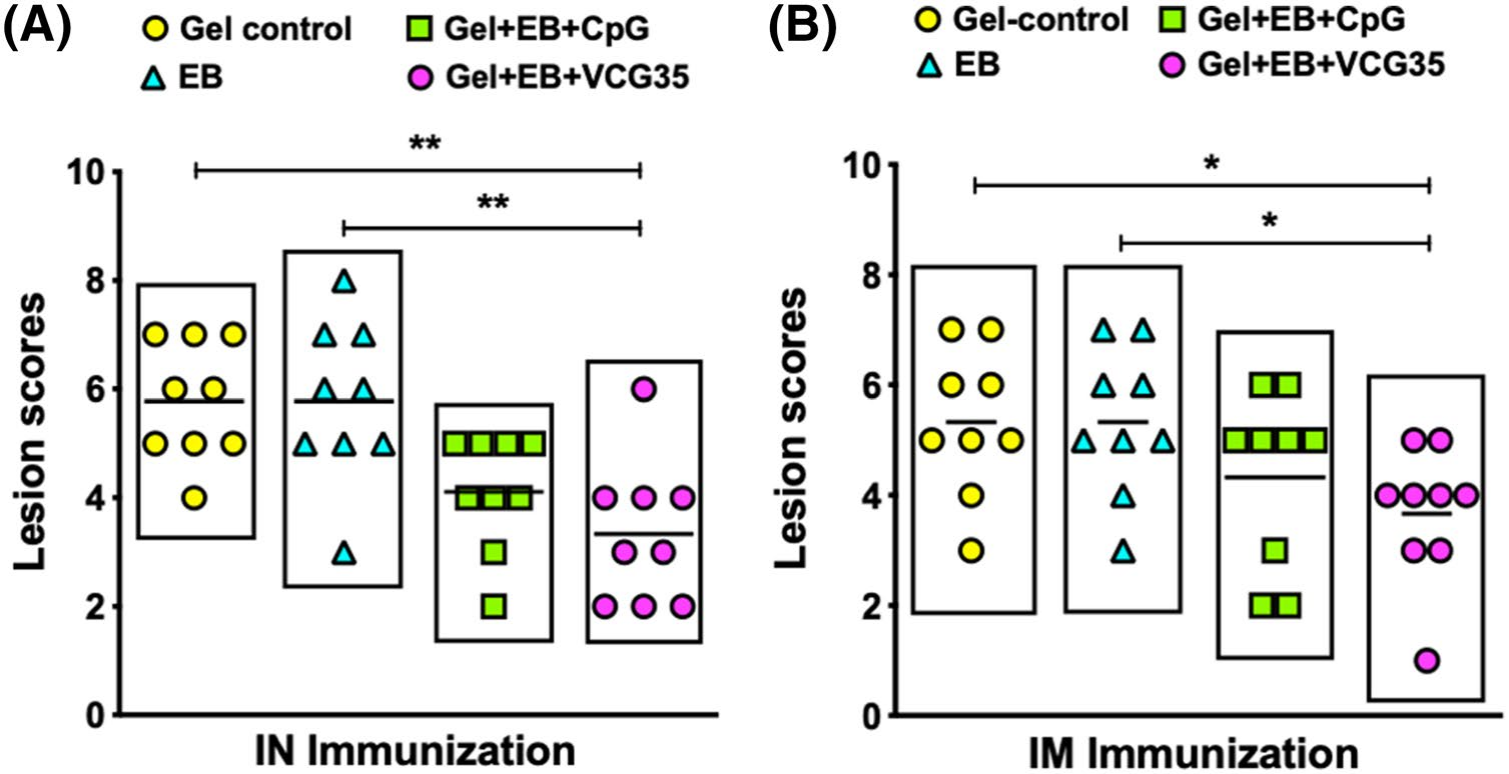
Comparison of lung and air sac pathological lesions. Groups of chickens immunized IN (**A**) or IM (**B**) as described above were challenged intranasally with 1 × 10^8^ IFU of live *C. psittaci* 2 weeks after the booster immunization. Chickens from each group (9/group) were euthanized 2 weeks post challenge, and harvested lungs and air sacs were macroscopically examined for pathological lesions and scored. The presence or absence of lesions and lesion severity were scored according to the criteria described in the text and the calculated total group severity score (the sum of the scores for lungs and air sacs) was recorded for each chicken. Differences between experimental groups were compared by One-way ANOVA with Tukey’s post multiple comparison test at p* < 0.05 and p** < 0.01.

Except for transient breathing difficulty observed for 3 days in the chickens immunized with EB and Gel alone control, there was no mortality in all immunized groups post challenge. Based on the criteria described in the Materials and Methods, the calculated total group lesion severity score, which is the sum of the scores for lungs and air sacs were recorded for each chicken. Irrespective of route of vaccine delivery, chickens in the Gel control and EB immunized groups had the most severe pathological lesions, which were significantly higher compared to those in the Gel + EB + VCG35 (*p* < 0.01) and Gel + EB + CpG (*p* < 0.05) vaccine groups (Fig. 5). Although lower lesion severity scores were associated with chickens vaccinated with Gel + EB + VCG35 compared to Gel + EB + CpG following both routes of immunization, the difference was not statistically significant (*p* > 0.05). Furthermore, lower lesion scores were associated with chickens vaccinated with the Gel + EB + VCG35 vaccine following IN mucosal compared to IM systemic delivery, although the difference was not statistically significant (*p* > 0.05), indicating both routes were equally effective in preventing lung/air sac lesions.

### IN mucosal immunization with EB formulated in chitosan gel in combination with different doses of VCG induced robust dose-dependent cellular and humoral immune responses in chickens

**Figure 6:**
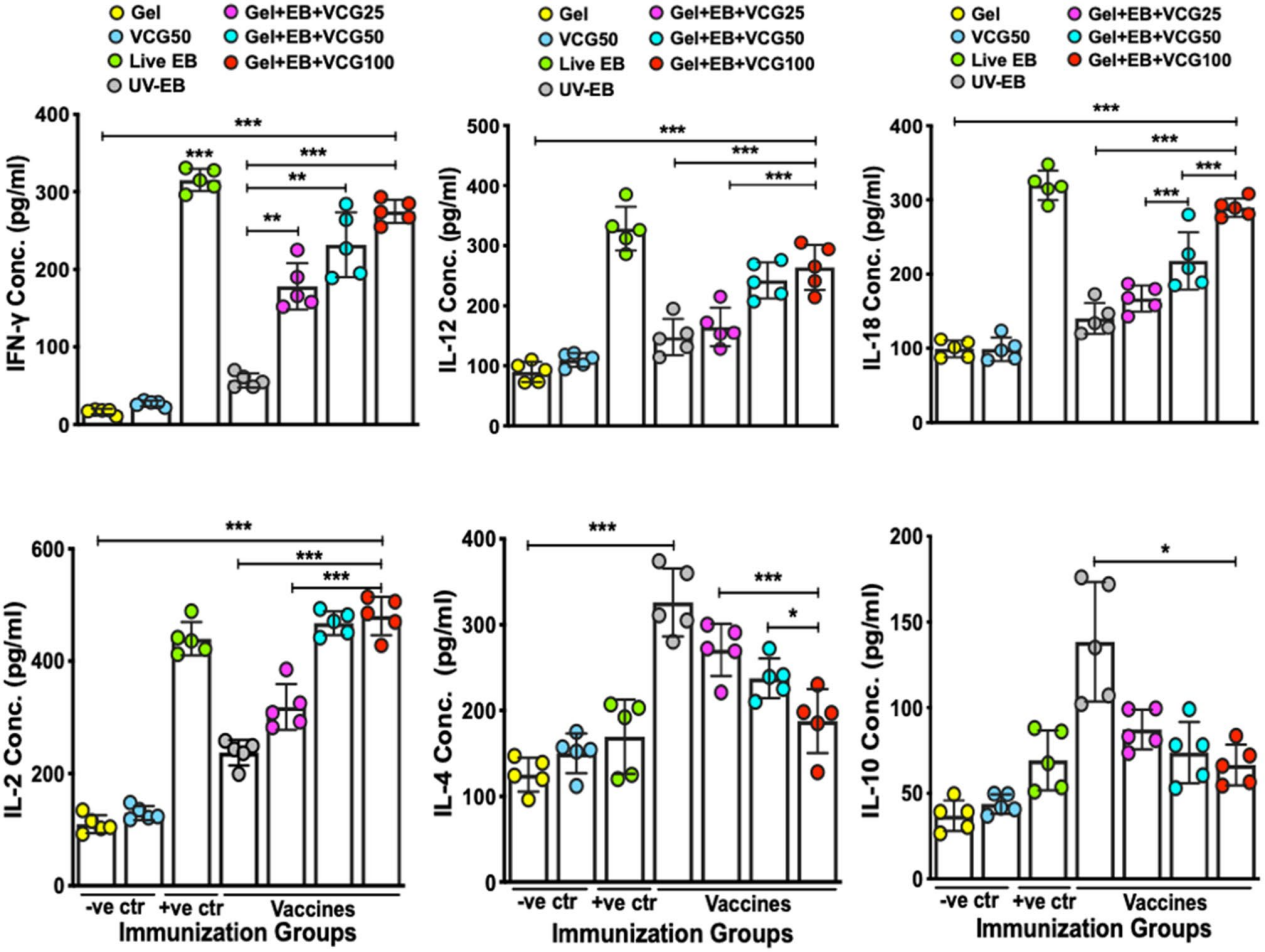
Concentration of cytokines in lung lavage fluids of chickens immunized with UV-inactivated EB in combination with different VCG doses. Groups of chickens (8/group) were immunized and boosted IN as described in the materials and methods section. Lung lavage fluids were obtained 3 weeks after the booster immunization. The amount of Th1 (IFN-γ, IL-2, IL-12, IL-18), Th2 (IL-4) and anti-inflammatory (IL-10) cytokines in supernatants of lung lavage fluids was quantified using commercial cytokine assay kits from Kingfisher (Kingfisher Biotech Inc, Saint Paul, MN) and Cusabio (Cusabio, Wuhan, China). The concentration of the cytokines in each sample was extrapolated from a standard calibration curve generated simultaneously. Bars represent the mean concentrations (± SD) from 8 chickens and are the results of one of two independent experiments with similar results. Significant differences between groups were evaluated by One-way ANOVA with Tukey’s post multiple comparison test at p*** < 0.001, p** < 0.01 and p* < 0.05. Abbreviations: − *ve ctr* negative controls; + *ve ctr* positive control.

Having established the superiority of mucosal over systemic immunization in inducing immune responses to the Gel + EB + VCG35 vaccine formulation in Expt. 1, we then sought to evaluate the effect of different concentrations of VCG on the magnitude of immune responses generated by mucosal immunization with EB formulated in chitosan gel. Since cellular immunity is a key component of the protective immune response following *Chlamydia* infection, we evaluated the magnitude of Th1 (IFN-γ, IL-2, IL-12, IL-18), Th2 (IL-4) and anti-inflammatory (IL-10) cytokines (Expt. 2) elicited in lung lavage fluid of chickens 3 weeks after IN booster immunization. The results showed IN mucosal immunization with VCG adjuvanted vaccines induced the secretion in lung lavage fluid of significantly higher (*p* < 0.05) levels of Th1 cytokines compared to EB or Gel control in a dose-dependent manner (Fig. 6). The results also showed immunization with the Gel + EB + VCG100 vaccine formulation stimulated the production of significantly higher (*p* < 0.01) levels of Th1-type cytokines (IFN-γ, IL-2, IL-12, IL-18) compared to the Th2 (IL-4) and anti-inflammatory (IL-10) cytokines. As expected, immunization with live *C. psittaci* EBs induced the secretion of significantly higher (*p* < 0.01) levels of antigen-specific Th1-type cytokines compared to EBs. In contrast, the amount of the IL-4 secreted in lung lavage fluid of chickens immunized with EB was significantly higher (*p* < 0.01) than that secreted in live *C. psittaci* EB-immunized chickens (Fig. 6). Also, levels of IL-10 were significantly higher (*p* < 0.01) in lung lavage fluid of chickens immunized with EB alone compared to EB co-delivered with VCG.

**Figure 7:**
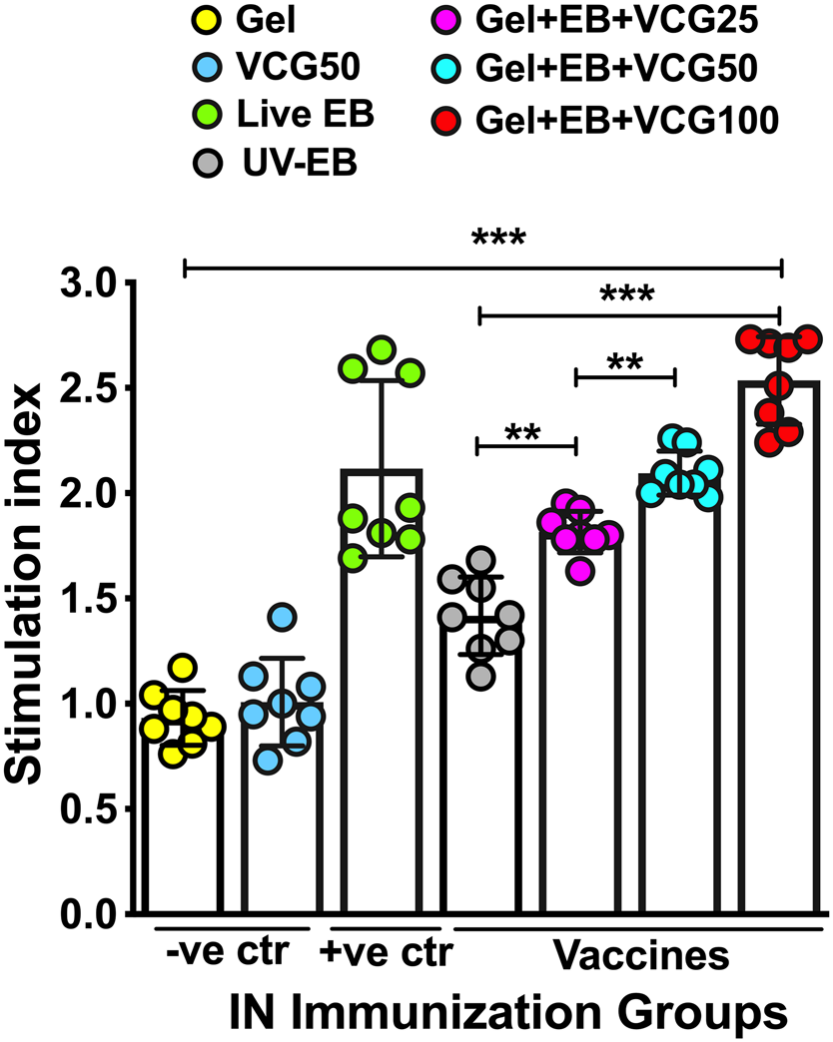
Proliferative responses of PBMCs from chickens immunized with UV-inactivated EB in combination with different doses of VCG. Groups of chickens (8/group) were immunized IN with two doses of the vaccine formulations and controls, 2 weeks apart. Three weeks after the booster immunization, PBMCs obtained by Lymphoprep density gradient centrifugation were restimulated in vitro with 10^6^ IFU of UV-irradiated *C. psittaci* EBs (*C. psittaci* antigen) for 24 h at 37℃ in 5% CO_2_. The antigen-specific proliferative response of PBMCs was determined using **the 5-Bromo-2’-deoxy-uridine (BrdU) cell proliferation assay kit (#K306; Abcam, Beijing, China); incorporation was detected by addition of TMB substrate and the optical density was read at 450 nm. Results are expressed as the stimulation index (SI), the ratio between absorbance values of stimulated and non-stimulated cells and the bars represent the mean and SD of three independent experiments. Significant differences between groups were evaluated by One-way ANOVA with Tukey’s post multiple comparison test at p*** < 0.001 and p** < 0.01. Abbreviations: − *ve ctr* negative controls; + *ve ctr* positive control.

We also assessed if the concentration of VCG will impact the magnitude of proliferation of PBMCs from immunized chickens. The magnitude of lymphocyte proliferation was expressed as stimulation index (SI), defined as the ratio of the absorbance values of stimulated and non-stimulated cells. We found that the proliferative responses of PBMCs to antigenic restimulation were dose dependent (Fig. 7), indicating that the concentration of VCG in the vaccine formulation influenced the magnitude of lymphocyte proliferation. These responses were significantly higher (*p* < 0.01) in all formulations incorporating VCG compared to EB alone, with the Gel + EB + VCG100 vaccine formulation showing the most significant difference (*p* < 0.001). Interestingly, mucosal delivery of the Gel + EB + VCG vaccine formulation elicited significantly higher (*p* < 0.01) proliferative responses compared to immunization with live *C. psittaci* EBs.

**Figure 8:**
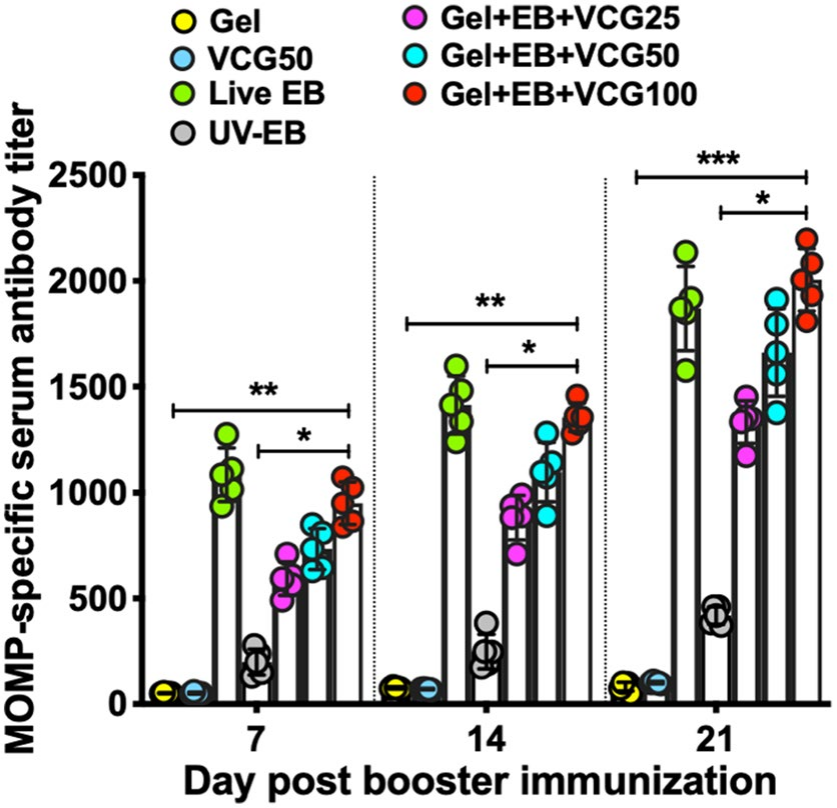
Detection of MOMP-specific antibodies after immunization with UV-inactivated EB in combination with different doses of VCG. Groups of chickens were immunized and boosted IN as described in the materials and methods section. Serum samples were collected from 5 chickens per group on days 7, 14 and 21 after the booster immunization as indicated in the Materials and Methods. MOMP-specific antibody titers were assessed by a MOMP-based antibody ELISA. Results are presented as the reciprocal of the highest serum dilution with an absorbance above the cut-off value (mean negative OD values plus 2 times the SD). The data are from one of two independent experiments with similar results. Significant differences between experimental groups were evaluated by One-way ANOVA with Tukey’s post multiple comparison test at p*** < 0.001, p** < 0.01 and p* < 0.05.

Immunized chickens developed MOMP-specific serum antibodies to VCG adjuvanted Gel + EB + VCG vaccine formulations, as indicated by the antibody titers, in a dose dependent manner on days 7, 14 and 21 post booster immunization (Fig. 8). The antibody titers elicited by the Gel + EB + VCG100 vaccine formulation were significantly higher (*p* < 0.01) compared to those elicited by EB alone or Gel control (Fig. 8). Moreover, the Gel + EB + VCG100 titers were comparable to those elicited after immunization with live *C. psittaci* EBs (positive control).

### The immune effectors elicited in response to mucosal immunization of chickens with Gel + EB + VCG100 afforded the most significant level of protection

**Figure 9:**
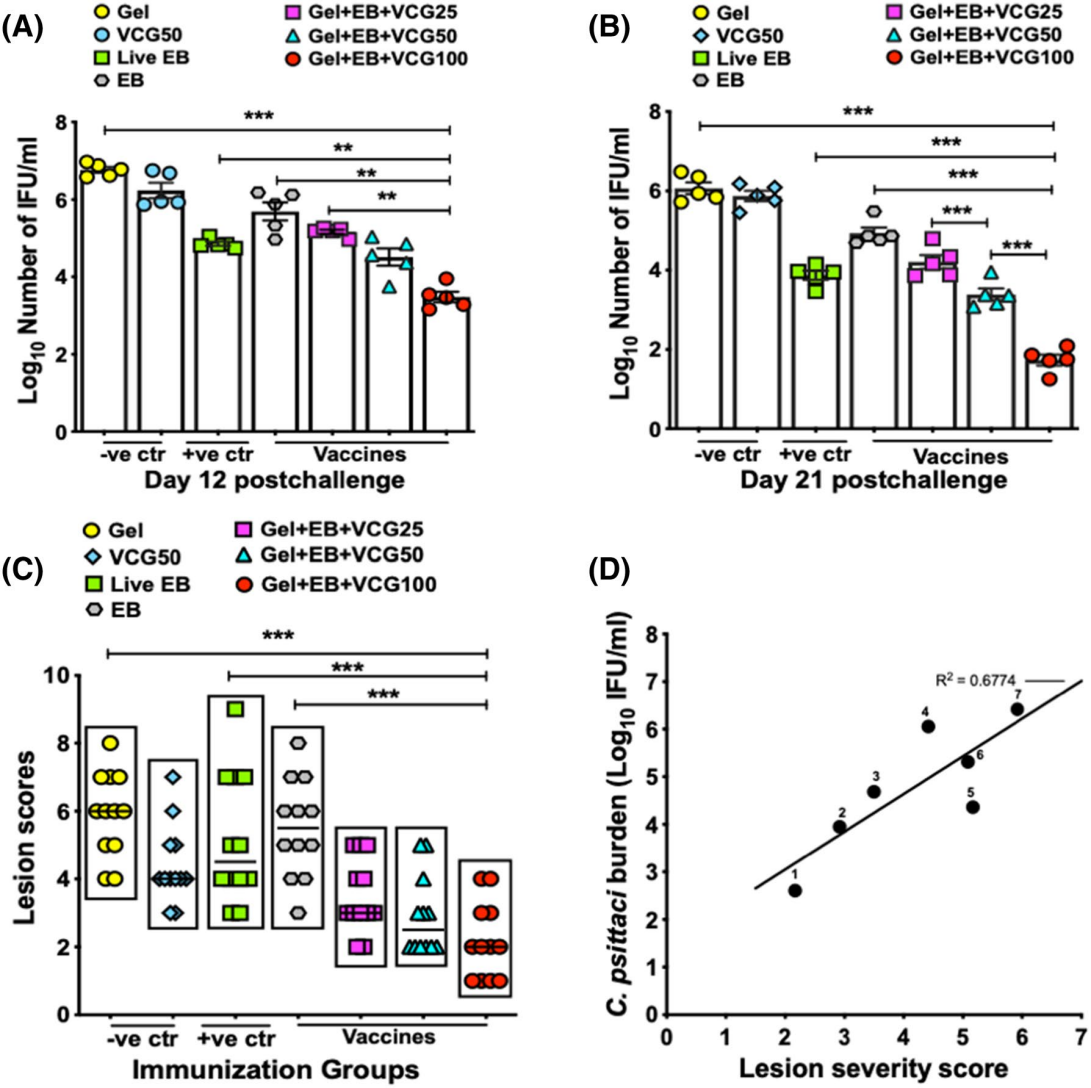
Evaluation of pharyngeal *C. psittaci* burden and pathological lesions in chickens immunized with UV-inactivated EB in combination with different doses of VCG, and relationship of pharyngeal *C. psittaci* burden to lesion severity. Groups of chickens were immunized and boosted IN as described in the materials and methods section. Immunized and control chickens were challenged 3 weeks after the booster immunization. Pharyngeal swabs were obtained from individual chickens (6/time point) on days 12 (**A**) and 21 (**B**) post challenge and *C. psittaci* was isolated from pharyngeal swabs in tissue culture. Chlamydial inclusions were detected by immunofluorescence using the Imagen immunofluorescence kit (Oxoid, Cambridge UK) and enumerated. The data is shown as the number of recoverable IFUs expressed as log_10_ IFU/ml per group. (**C**) Lungs and air sacs were harvested from immunized chickens (12/group) 3 weeks after challenge and gross pathological lesions were evaluated and scored. The total lesion severity scores were then calculated and recorded for each chicken. The data is shown as the calculated total lesion severity scores (the sum of the scores for lungs, thoracic and abdominal air sacs) of individual chickens. Differences between experimental groups were compared by One-way ANOVA with Tukey’s post multiple comparison test at p*** < 0.001 and p** < 0.01. Abbreviations: − *ve ctr* negative controls; + *ve ctr* positive control. (**D**) The mean lesion severity scores and mean number of IFU were calculated and plotted for each vaccine group. Data are combined from days 12 and 21 postchallenge. Pharyngeal *C. psittaci* burden values are plotted along the Y-axis, and the lungs/air sacs lesion severity scores are plotted along the X-axis. Each dot represents a single immunization group. 1, Gel-EB-VCG100; 2, Gel-EB-VCG50; 3, Gel-EB-VCG25; 4, VCG50; 5, Live EB; 6, EB alone; 7, Gel alone. The correlation between lesion severity and pharyngeal *C. psittaci* burden was evaluated by Spearman’s correlation coefficient; R^2^ = 0.6774. Abbreviations: − *ve ctr* negative controls; + *ve ctr* positive control.

Having established that the Gel + EB + VCG100 formulation elicited the most robust immune effectors compared to the other antigen formulations studied, we then investigated if these immune effectors could protect against pharyngeal *C. psittaci* infection and prevent development of lung and air sac lesions following intranasal challenge with live *C. psittaci* EBs. Protection against infection, assessed by pharyngeal *C. psittaci* clearance, showed the effect of VCG on chlamydial burden was dose dependent. By day 12 post challenge, the pharyngeal *C. psittaci* load in Gel + EB + VCG100 immunized chickens was about 3.2-log lower than the Gel control group and 2.2-log lower than the EB immunized group (Fig. 9A). Also, chickens immunized with live EBs shed significantly (*p* < 0.05) fewer *C. psittaci* IFUs when compared to those immunized with EB. By day 21 post challenge, there was a further reduction in pharyngeal *C. psittaci* load in chickens immunized with VCG-containing formulations, with the Gel + EB + VCG100 formulation showing a significant protective advantage (Fig. 9B).

With regards to clinical symptoms, except for transient breathing difficulty in chickens immunized with EB, Gel and VCG50 control groups, no mortality or severe respiratory symptoms were observed following challenge. On the basis of the scoring criteria described above, the calculated total lungs and air sacs lesion severity scores of chickens immunized with the VCG containing formulations were significantly (*p* < 0.001) lower compared to chickens immunized with EBs, live EBs or Gel controls. While protection against respiratory pathology by the VCG containing formulations was dose dependent, the Gel + EB + VCG100 vaccine formulation was the most effective in protecting against lung and air sac lesions (Fig. 9C). Examination of the protection results suggested that the *C. psittaci* burden corresponded with the severity of the respiratory pathology lesions. We therefore evaluated if the *C. psittaci* burden correlated with the pathology severity scores. Linear regression analysis of the two protective parameters showed moderate correlation (Spearman’s correlation coefficient; R^2^ = 0.6774) (Fig. 9D). Taken together, the results indicate that mucosal immunization of chickens with Gel + EB + VCG100 afforded the most significant level of protection against both *C. psittaci* infection and development of pathology after challenge.

## Discussion

Inactivated whole *Chlamydia* EBs do not usually provide significant protection against infection, but recent studies show that they could be modulated to induce significant protective immunity by modifying the delivery system or by combination with appropriate adjuvants^37^. Oral delivery is the most preferred route of drug administration; it is safe, convenient, cost-effective, needle-free, universally acceptable, easy to administer without need for specialized training. However, certain issues such as gastric irritation and varied bioavailability have prevented its universal application especially in vaccine delivery. To overcome these drawbacks, the intranasal route is considered an effective alternative as it is relatively easy, noninvasive and increases bioavailability. In addition to being needle-free, intranasal delivery will have the ability to induce local immune responses directed to the site of pathogen entry. Even so, the IM route has been shown to be capable of inducing protective immunity at both systemic and mucosal tissues^48^.

In this study, we tested the hypothesis that UV-inactivated *C. psittaci* EB formulated in VCG, which are empty *V. cholerae* cell envelopes in combination with chitosan nanoparticles will induce robust protective immunity against intranasal challenge in SPF chickens. Based on the results of our preliminary studies and data from published works, we opined that the use of chitosan as a delivery vehicle may enhance the adjuvant capacity of VCG. First, we compared the impact of VCG and CpG adjuvants on protective immunity following IN mucosal and IM systemic delivery of EB formulated in chitosan hydrogel. Immunologic evaluation revealed that the IN route of immunization induced higher levels of the Th-1-type cytokines IFN-γ, IL-2 and IL-12 compared to the IM route. In addition, the finding that mucosal and systemic delivery of EB with VCG activated more robust antigen-specific IFN-γ production and proliferation of PBMCs compared to CpG confirms the results of our previous in vitro dendritic cell (DC) studies^57,65^, which established the superior adjuvanticity of VCG over CpG. It is known that protection against *Chlamydia* is mediated primarily via the induction of CD4+ and CD8 + T cells secreting high levels of IFN-γ^15^. Previous studies in both mice and humans showed that CD4 + T cell-dependent mechanisms, involving IFN-γ secretion and induction of inducible nitric oxide synthetase (iNOS) play a central role in protection against *C. muridarum* genital infection^66,67^. Our previous studies in mice using *C. trachomatis* confirm that vaccine regimens, which afford protection against challenge also stimulate CD4 + T cells that secrete high levels of the Th1 type cytokine, IFN-γ^35,48^. However, other studies indicate that CD8 + T cells may play a more crucial role than CD4 + T cells in protection against *C. psittaci*^17^*.* We also noted that lung lavage fluids of chickens immunized with EB alone secreted significantly higher levels of the anti-inflammatory cytokine, IL-10 compared to EB in combination with either VCG or CpG, irrespective of route of vaccine delivery. IL-10 is a cytokine produced by both innate and adaptive immune cells, including Th1, Th2, and Th17 T cell subsets, Treg cells, CD8 + T cells, and B lymphocytes and plays an important role in regulating inflammatory immune responses^68,69^. It is a potent anti-inflammatory cytokine whose role is to repress the normal host inflammatory response during infections^70^. We could not assay for levels of the Th-2-type cytokines IL-4 or IL-13 due to the unavailability of these chicken cytokines and therefore could not determine if the observed cellular responses were of Th1- or Th2.

We observed a significant increase in MOMP-specific antibody titers following both IN and IM immunization when compared to the controls, indicating the ability of inactivated EB in combination with either VCG or CpG to elicit substantial humoral immune responses following both mucosal and systemic delivery. While the role of CD4+ and CD8 + T cell-dependent mechanisms in protection against *Chlamydia* has been established^17^^,^^71^, the relative contribution of antibody in the resolution of a primary chlamydial infection is still unfolding. Thus, although there was no difference in the course of a primary genital chlamydial infection in B‐cell‐deficient and control mice, the former suffered more intense disease upon reinfection than the control mice^72^. Furthermore, B‐cell‐deficient mice depleted of CD4^+^ T cells were incapable of controlling a secondary genital chlamydial infection^22^. However, recent studies suggest that the predominant role of antibodies in chlamydial clearance is in resistance to re-infection by enhancing the uptake, processing, and presentation of chlamydial antigens by antigen presenting cells for rapid Th1 activation and clearance of infection^24,73^. Also, it has been suggested that antibody-mediated immunity may be due to the recruitment and/or activation of an accessory non-T cell population that functions with antibody to resolve infection and protect against reinfection^73^.

Efficacy analyses of the EB formulations against challenge infection with live *C. psittaci* revealed that the group of chickens immunized IN with the Gel-EB formulated with VCG showed enhanced pharyngeal bacterial clearance. These animals shed about 2-log lower IFUs than the Gel control-immunized chickens. Specificity of vaccine efficacy is addressed by the inclusion of Gel alone (carrier) control. Chickens immunized with EB alone were not protected. Various studies in mice have shown immunization with *C. trachomatis* EB does not afford substantial protective immunity^37,57^. Further vaccine efficacy analysis showed chickens immunized with EB adjuvanted with VCG and CpG were significantly protected from development of severe lung and air sac lesions with immunization via the IN mucosal route showing a protective advantage. Similar to chickens immunized with Gel alone, chickens immunized with EB alone were not protected from development of pathological lesions. These studies reveal that formulation of inactivated chlamydial antigens with certain adjuvants increases their ability to induce cellular and humoral immune responses accompanied by protection against challenge, and ability to prevent or reduce development of pathology. Thus, in terms of efficacy, defined by reduction in pharyngeal *C. psittaci* burden and protection against lung and air sac lesions following immunization with EB adjuvanted with VCG35 and CpG vaccines, we conclude that mucosal immunization is superior to systemic delivery.

After establishing the superiority of mucosal over systemic delivery of EB and the adjuvant advantage of VCG over CpG, we then evaluated the impact of different concentrations of VCG on protective immunity. The impact of VCG on the magnitude of immune responses to immunization was evaluated by comparing the cellular and humoral immune responses elicited following immunization with the vaccines containing different concentrations of VCG. The observation of significantly elevated IFN-γ over IL-4 levels in lung lavage fluids of immunized chickens indicates the recruitment and retention of Th1 cells in the respiratory secretions of mucosally immunized chickens. Vaccine efficacy evaluation was based on the combination of pharyngeal *C. psittaci* burden as a function of time post-challenge and lesion severity scores. Specificity of the efficacy of the vaccines was addressed by the inclusion of Gel alone (carrier) and VCG50 alone (adjuvant) negative controls as well as live *C. psittaci* as a positive control. We demonstrated that mucosal immunization induced robust dose-dependent cellular and humoral immune responses that significantly reduced pharyngeal shedding and protected immunized chickens from severe lung and air sac pathological lesions. Our observation that pharyngeal *C. psittaci* burden only moderately correlated with pathological lesion scores underlines the need for vaccine efficacy evaluation to include monitoring protection against infection and inflammation-induced pathology^74^. Previous studies established the effectiveness of the *Vibrio cholerae* ghost (VCG) platform for stimulating robust protective immunity even in the absence of other adjuvants^47–49^. Also, studies in our laboratory highlighted the potential of incorporating mucosal adjuvants in enhancing protective immunity^35,75^. These studies, employing mucosal and systemic immunization routes and a variety of chlamydial vaccine antigens, highlighted the significance of route of vaccine administration in the induction of protective immunity^35,48^.

In conclusion, we have shown that the efficacy of inactivated chlamydial antigens, otherwise poorly immunogenic, can be improved by co-delivery with adjuvants. We also demonstrated the superiority of mucosal over systemic delivery of VCG-adjuvanted inactivated *C. psittaci* EBs, indicating that route of vaccine delivery is an important determinant of the type and potency of immune responses elicited by vaccination with diverse antigens. We further showed the adjuvanticity of VCG was concentration-dependent, since protective immunity induced following IN mucosal immunization showed dose-dependent immune responses and protection. The significant reduction in pharyngeal shedding, as indicated by the number of recoverable *C. psittaci* IFUs and reduction in lung and air sac lesions by the VCG vaccine formulations underlines the potential of VCG as a vaccine adjuvant. Further studies will establish the optimum dose of VCG that will induce maximum protective immunity.

## Supplementary Information

Supplementary Information.
