# Supplementary material for: Intranasal immunization with inactivated chlamydial elementary bodies formulated in VCG-chitosan nanoparticles induces robust immunity against intranasal Chlamydia psittaci challenge
Source: Sci Rep. 2021 May 17;11:10389. doi: 10.1038/s41598-021-89940-8 (PMC8129140; doi:10.1038/s41598-021-89940-8)
Supplement: Supplementary file 1 — Supplementary Information. [file 41598_2021_89940_MOESM1_ESM.pdf]

Supplementary Material

Intranasal immunization with inactivated chlamydial elementary bodies formulated in VCG-chitosan nanoparticles induces robust immunity against intranasal *Chlamydia psittaci* challenge

Zonghui Zuo, Yongjuan Zou, Qiang Li, Yongxia Guo, Tianyuan Zhang, Jie Wu, Cheng He* and Francis O. Eko*

* Corresponding authors: feko@msm.edu; hecheng@cau.edu.cn

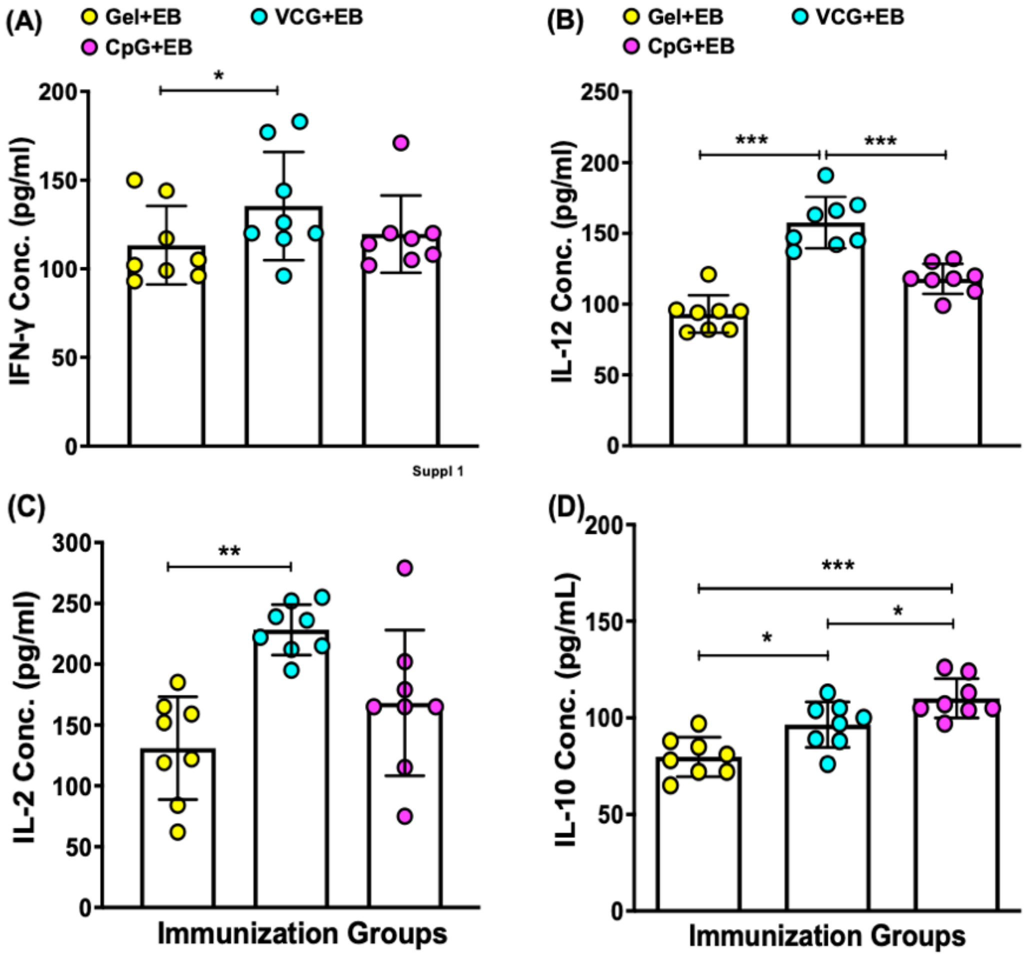

Supp Fig. 1. Induction of *Chlamydia psittaci*-specific cytokines in lung lavage fluid after immunization with Gel+EB, VCG+EB and CpG+EB. Groups of chickens (10/group) were immunized and boosted IN, 2 weeks apart. The concentration of cytokines; IFN-γ (A), IL-12 (B), IL-2 (C) and IL-10 (D) contained in lung lavage fluids from immunized chickens was measured by cytokine ELISA assay kits from Kingfisher (Kingfisher Biotech Inc, Saint Paul, MN) 2 weeks after the booster immunization. The concentration of the cytokines in each sample was obtained by extrapolation from a standard calibration curve generated simultaneously. Data were calculated as the mean values (± S.D.) for triplicate wells for each assay. Significant differences between experimental groups were evaluated at $p^* < 0.05$, $p^{**} < 0.01$ and $p^{***} < 0.001$.

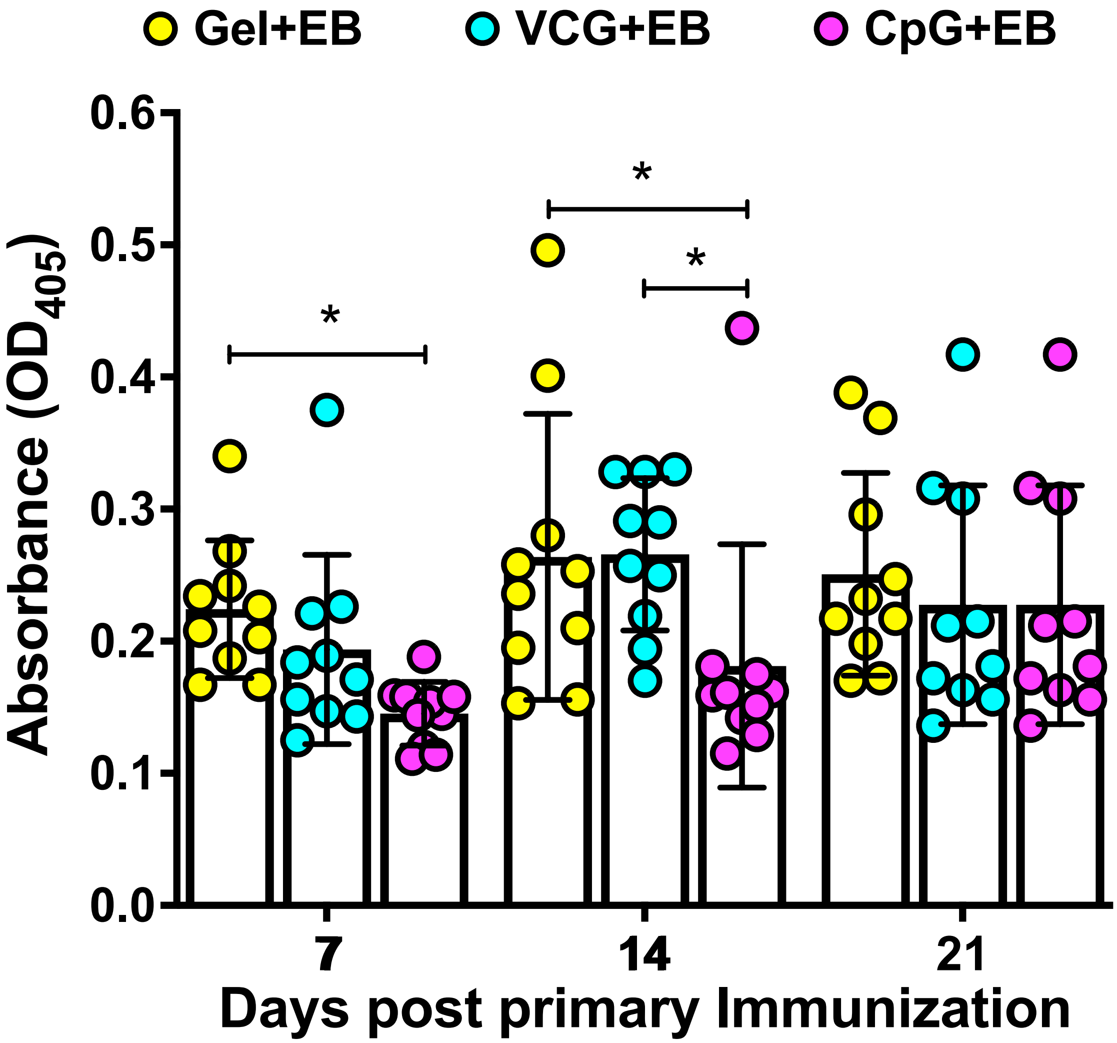

Supp Fig. 2. MOMP-specific antibodies elicited after immunization with Gel+EB, VCG+EB and CpG+EB. Groups of chickens (10/group) were immunized IN twice, 2 weeks apart and serum samples were obtained on days 7, 14 and 21 post primary immunization. A MOMP-based antibody ELISA was used to compare the level of antibodies elicited in serum of immunized chickens. The optical density of the wells was read at 405 nm and the data are presented as raw absorbance values (OD_{405}). The results are from one of two independent experiments with similar results. Significant differences between experimental groups were evaluated at ($p^* < 0.05$).

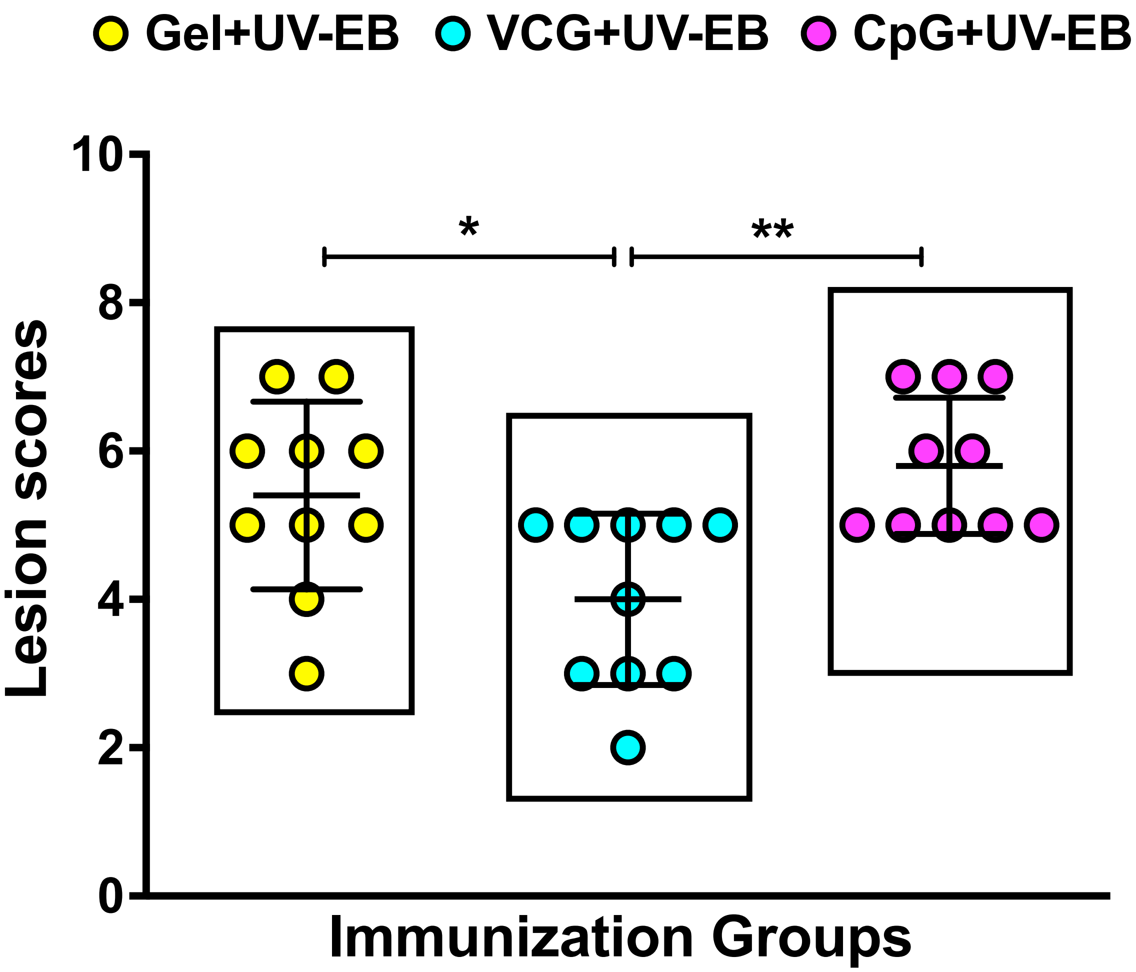

Supp Fig. 3. Comparison of lung and air sac pathological lesions after immunization with Gel+EB, VCG+EB and CpG+EB. Groups of chickens (10/group) immunized IN twice, 2 weeks apart were challenged intranasally with 1 x10^8 IFU of live *C. psittaci* 3 weeks after the last immunization. Two weeks post challenge, the lungs, thoracic and abdominal air sacs were harvested and macroscopically examined for pathological lesions and scored. The data is shown as the total group severity score (the sum of the scores for lungs, thoracic and abdominal air sacs) recorded for each chicken. Differences between experimental groups were compared by Kruskal–Wallis One-way ANOVA at p*< 0.05 and p* < 0.01.

Kingfisher biotech

IL-2

conc.(pg/ml)	1000	500	250	125	62.5	31.25
OD mean (two replicates)	2.2355	1.1035	0.7595	0.436	0.3675	0.2335

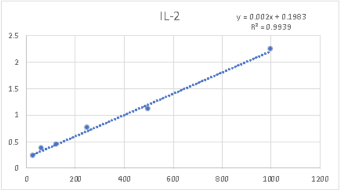

IL-12

conc.(pg/ml)	1000	500	250	125	62.5	31.25
OD mean (two replicates)	0.2686	0.1061	0.0611	0.0216	0.0556	0.0251

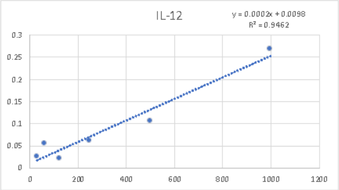

IFN-r

conc.(pg/ml)	1000	500	250	125	62.5	31.25
OD mean (two replicates)	0.088	0.0355	0.025	0.018	0.0145	0.011

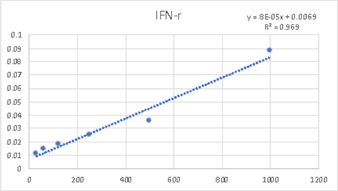

IL-10

conc.(pg/ml)	1000	500	250	125	62.5	31.25
OD mean (two replicates)	0.1445	0.0605	0.034	0.008	0.006	0.00455

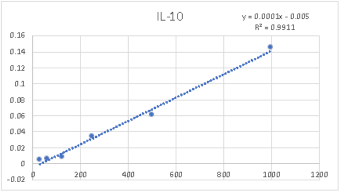

CUSABIO

IL-4

conc.(pg/ml)	800	400	200	100	50	25	12.5
OD mean (tw	1.179	0.939	0.778	0.522	0.467	0.323	0.189

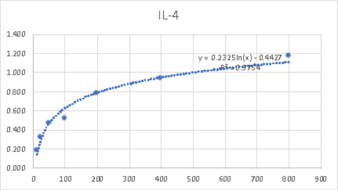

IL-18

conc.(pg/ml)	1000	500	250	125	62.5	31.2	15.6
OD mean (tw	1.905	1.619	1.307	1.042	0.711	0.393	0.375

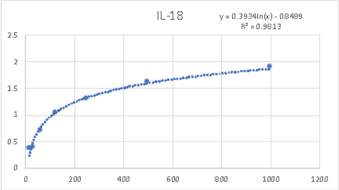

Supp Fig. 4. Reference lines (standard curves) for cytokine ELISAs. The concentration of cytokines in each sample was extrapolated from a standard calibration curve generated simultaneously.

Step	IFN-γ ELISA		IL-2 ELISA		IL-12 ELISA		IL-10 ELISA	
	Amount	Duration	Amount	Duration	Amount	Duration	Amount	Duration
**Coating** (Capture Antibody)	PB0442C-100 5 µg/mL, 100 µL	Overnight, RT	PB0387C-100 2.5 µg/mL, 100 µL	Overnight, RT	PB0435C-100 2.5 µg/mL, 100 µL	Overnight, RT	KP1116C-100 5 µg/mL, 100 µL	Overnight, RT
**Blocking**	1% Non-fat dry milk, 100 µL	1 hour, RT	1% Non-fat dry milk, 100 µL	1 hour, RT	1% Non-fat dry milk, 100 µL	1 hour, RT	1% Non-fat dry milk, 100 µL	1 hour, RT
**Highest standard**	RP0115C-005 1000 pg/mL	2 hours, RT	RP0063C-005 1000 pg/mL	1 hour, RT	RP0289C-005 1000 pg/mL	1 hour, RT	RP0018C-005 1000 pg/mL	2 hours, RT
**Lowest standard**	31.25 pg/mL, 100 µL		31.25 pg/mL, 100 µL		31.25 pg/mL, 100 µL		31.25 pg/mL, 100 µL	
**Sample**	300 µL		100 µL		100 µL		100 µL	
**Biotinylated secondary antibody**	PBB0448C-050 0.25 µg/mL, 100 µL	1.5 hours, RT	PBB0395C-050 0.1 µg/mL, 100 µL	1 hour, RT	PBB0436C-050 0.1 µg/mL, 100 µL	1 hour, RT	KPB1117C-050 0.1 µg/mL, 100 µL	1 hour, RT
**Streptavidin -HRP**	1:100, 100 µL	30 min, RT	1:100, 100 µL	30 min, RT	1:100, 100 µL	30 min, RT	1:100, 100 µL	30 min, RT
**Develop with TMB**	100 µL	30 min, RT	100 µL	30 min, RT	100 µL	30 min, RT	100 µL	10 min, RT
**TMB stop solution**	100 µL		100 µL		100 µL		100 µL	

Supplementary Table1: Protocols for chicken cytokine ELISA assays.
